# Artificial Intelligence Technologies and Practical Normativity/Normality: Investigating Practices beyond the Public Space

**DOI:** 10.12688/openreseurope.16536.2

**Published:** 2024-03-11

**Authors:** Ingvild Bode, Hendrik Huelss

**Affiliations:** 1 Department of Political Science and Public Administration, Syddansk Universitet, Odense, Region Syddanmark, Denmark

**Keywords:** artificial intelligence, governance, norms, practices, technologies, International Relations, military, weapon systems

## Abstract

This essay examines how artificial intelligence (AI) technologies may shape international norms. Following a brief discussion of the ways in which AI technologies pose new governance questions, we reflect on the extent to which norm research in the discipline of International Relations (IR) is equipped to understand how AI technologies shape normative substance. Norm research has typically focused on the impact and failure of norms, offering increasingly diversified models of norm contestation, for instance. But present research has two shortcomings: a near-exclusive focus on modes and contexts of norm emergence and constitution that happen in the public space; and a focus on the workings of a pre-set normativity (ideas of oughtness and justice) that stands in an unclear relationship with normality (ideas of the standard, the average) emerging from practices. Responding to this, we put forward a research programme on AI and practical normativity/normality based on two pillars: first, we argue that operational practices of designing and using AI technologies typically performed outside of the public eye make norms; and second, we emphasise the interplay of normality and normativity as analytically influential in this process. With this, we also reflect on how increasingly relying on AI technologies across diverse policy domains has an under-examined effect on the exercise of human agency. This is important because the normality shaped by AI technologies can lead to forms of non-human generated normativity that risks replacing conventional models about how norms matter in AI-affected policy domains. We close with sketching three future research streams. We conclude that AI technologies are a major, yet still under-researched, challenge for understanding and studying norms. We should therefore reflect on new theoretical perspectives leading to insights that are also relevant for the struggle about top-down forms of AI regulation.

## Plain language summary

This essay critically discusses the interplay between artificial intelligence (AI) technologies and norms, focusing on the discipline of International Relations (IR). Here, norms are usually conceptualised as the products of reflection processes where actors debate and decide on “agreeable” standards. IR research on AI governance therefore turns towards international public and deliberative processes and their outcomes. But this risks missing two crucial pathways of how AI technologies may shape norms: first, how practices of designing and using AI technologies performed by actors at sites outside of the public eye make norms; and second, how design and use AI technologies may contribute to not only shaping normativity, in the sense of ideas of oughtness and justice, but also normality, in the sense of the average and the normal. We conclude that AI technologies pose an important challenge for research on norms and consequently require new conceptual and regulative perspectives.

## Introduction

Artificial intelligence technologies (AITs) have gained increasing media attention, in particular since the roll-out of so-called generative forms of AI systems, such as ChatGPT that produce text based on probabilistic prediction of “characters, words, or sentences in a sequence” ( Tarnoff, 2023). In response, the political and popular mood appears to fluctuate between a techno-optimist infatuation with the purported dramatic advances of ‘AI’ and techno-sceptical concerns with its societal risks, including worrying failures. At the moment of writing (February 2024), AI technologies remain lightly regulated but the growing number of applications based on AI technologies result in major governance demands for national, regional, and international policymakers to address ( Paul, 2022; Tallberg *et al.*, 2023). The emerging governance and regulation regime of AI technologies takes various forms, with both general and policy-field specific approaches being sought by public and private actors – or a combination thereof ( Veale *et al.,* 2023). AI governance outcomes sought after may be of a formal type, such as legislation or the creation of dedicated international institutions ( Maas & Villalobos, 2023), but also include informal principles and suggestions. Internationally and regionally, governance processes associated with, chiefly, public actors have already produced outputs, such as *UNESCO’s Recommendation on the Ethics of AI* (2019) and the *OECD’s Recommendation on AI* (2019). Another noteworthy example is the Group of Governmental Experts (GGE) on emerging technologies in the area of lethal autonomous weapon systems (LAWS). Working in the context of the United Nations Convention in Certain Conventional Weapon Systems (CCW), the GGE on LAWS has provided a forum for states to discuss challenges associated with integrating AI technologies into weapon systems since 2017. On a regional scale, the European Union (EU) has agreed on its *Artificial Intelligence Act* in 2023, which looks set to become the first set of regional, legally enforceable rules *specifically pertaining* to AI technologies. Further, the Council of Europe’s Committee on AI is working to develop a legal instrument, which is publicly available in draft form (December 2023, Draft Framework Convention on AI, Human Rights, Democracy and the Rule of Law).

These diverse governance efforts offer scholars of norm research in International Relations (IR) manifold opportunities to study forums and processes where actors *publicly* express what kind of (governance) challenges they associate with AI technologies and begin to frame new norms, for example the EU’s vision of human-centric AI that is central to the *AI Act* (e.g. Amariles & Baquero, 2023; Carmel & Paul, 2022; Lukowicz, 2019). Norm research in IR has traditionally conceptualised norms as closely linked to international law. Early constructivist scholars, who introduced the empirical study of norms to the discipline, saw the institutionalisation of norms into soft or hard law as simultaneously the goal and the endpoint of norm emergence. Building on that, such scholarship focused on the specifics of state compliance with legally institutionalized norms via processes of diffusion, internalisation, and socialisation ( Finnemore & Sikkink, 1998; Keck & Sikkink, 1998; Risse *et al.,* 1999).

Later work in critical norm research has accommodated the inherent flexibility of norms by emphasising enacted ‘meaning-in-use’ ( Wiener, 2009; Wiener, 2017) across regions and contexts, focusing therefore on the much more dynamic and interactive processes of localisation, implementation, and contestation ( Acharya, 2009; Acharya, 2004; Betts & Orchard, 2014; Deitelhoff & Zimmermann, 2018; Drubel & Mende, 2023; Wiener, 2014; Zimmermann, 2017). Importantly, this line of research challenges a supposed linearity of norm development, as well as the questions of who has agency therein and whose agency counts ( Bucher, 2014; True & Wiener, 2019; Wiener, 2018). This scholarship also explores the very production of normativity in contestation processes ( Hofferberth & Weber, 2015; True & Wiener, 2019; Wiener, 2018), understanding contestation as a pivotal, norm-generative societal practice ( Orchard & Wiener, 2022, 54). These studies often remain connected/attached to soft or hard legally institutionalised norms or the public-deliberative process of drafting such norms and have produced many valuable insights.

But what such scholarship arguably does not account for is how the slow-moving nature of public-deliberative governance processes interacts with the, at times, fast pace of technological development, especially in (but not limited to) the field of ‘AI’. Even though several AI governance initiatives have yielded preliminary results in the shape of soft international law (e.g. sets of principles and non-binding resolutions), many of them are only moving forward slowly and often need re-adjusting to keep in step with technological change. While the EU’s AI Act has benefited from regulatory momentum, it still moves slowly through the EU’s institutions with more than two years passing between the presentation of its initial draft by the European Commission in April 2021 to its adoption by the European Parliament in June 2023. Further deliberations in EU member states create additional friction. The GGE on LAWS remains at a discussion rather than at a negotiation stage and insiders have invariably described its pace as glacial or even tectonic ( Régimbal, 2023). At the same time, applications utilising AI technologies are spreading across various aspects social life and public policy. Such technologies, for example, play a role in the provision of medical care such as radiography ( Obermeyer *et al.*, 2019) hiring decisions ( Ajunwa *et al*., 2016) to predictive policing ( Lum & Isaac, 2016) and the approval of welfare beneficiaries ( Eubanks, 2019). This development raises crucial regulatory questions because of the societal harms that such automated and algorithmic ‘solutions’ can produce, often reinforcing existing power asymmetries and hierarchies ( Birhane, 2021b). Partly due to a perceived lack of expertise, partly due to concerns about stifling innovation (see UK Government, 2023), the governance of AI is cumbersome and dominated by the input provided by a narrow set of dominant stakeholders, including major tech corporations ( Holland Michel, 2023, 4; Veale *et al.,* 2023).

This essay intervenes in the debate about governing AI by reflecting on governance through international norms, which we define as understandings of appropriateness ( Bode & Huelss, 2022, 12). ^1^ We put forward two analytical additions to norm research in the discipline of IR, and, in particular, to how norms emerge in IR, with the ambition of shaping a research programme on *practical normativity/normality*. (1) We focus on how, often operational, practices of designing and using AI technologies that are performed by actors *at sites outside of the public eye* become sources of normative substance on ‘AI’. This departs from a present focus in IR norm research on practices that are *observably* performed in public spaces, either in the form of “deliberation”, ^2^ i.e. how actors publicly discuss, consider, and verbalise norms, or via behavioural contestation ( Stimmer & Wisken, 2019; True & Wiener, 2019). But norms are not only constituted via practices associated with the public and/or deliberative space where they may transition into law, but also unfold in operational practices of design and use that are not subject to public debate, discursive codification, or public performance. This argument builds on scholarship at the theoretical interface of IR norm research and practice theories ( Bode & Karlsrud, 2019; Bode, 2023b; Gadinger, 2022; Laurence, 2018; Pratt, 2022; Ralph & Gifkins, 2017; Wiener, 2022), but also draws from critical security studies, as well as constructivist strands of science and technology studies (STS). Approaches that merge practice theories and norm research typically emphasize the relational, unstable dimension of norms, evidenced by their preference for concepts such as “normative configuration” or “normative substance” as alternatives to the seemingly well-bounded ‘norm’ ( Bode, 2024; Pratt, 2020; Pratt, 2022; Qiao-Franco & Bode, 2023).

(2) We consider how practices of designing and using AI technologies shape normative substance in an interplay between normativity, i.e. notions of moral duty and justice, and normality, i.e. notions of the typical and average ( Huelss, 2017). IR norm research that focuses on publicly performed practices often, implicitly rather than explicitly, emphasises the dimension of normativity. By contrast, the dimension of normality and the interactive relationship between the two concepts have only received little dedicated attention ( Gholiagha *et al.*, 2021; Huelss, 2020). However, both aspects are important to perceive and understand “previously obscured patterns and processes” ( Bernstein & Laurence, 2022, 77) in the relationship between norms and practices, especially when it comes to AI technologies. Our distinction between normativity and normality is ideal-typical/analytical in nature – separating between these two dimensions in empirical practice is typically infeasible because they can be likened to two sides of the same coin. At the same time, this also implies that it is crucial to conceptualise the relationship not least for analytical reasons. We will use the descriptor normativity/normality and interchange which of two notions we start off with. Notwithstanding this methodological challenge, we consider paying attention to both normality/normativity as vital and missing component of understanding the significance and reach of norms.

Our arguments are abductively informed by our empirical study and analysis of military applications of AI, in particular, of how AI and autonomous technologies have become incorporated into weapon systems. This development is typically connected to the catch-all term autonomous weapon systems (AWS) as systems that can ‘make’ targeting ‘decisions’ without immediate human intervention. ^3^ Targeting algorithms, for example, introduce forms of non-human centred ‘decision-making’ into the conduct of warfare.

Before we proceed, let us define what we mean by ‘AI’ and associated terminology. We use the term ‘AI’ to refer to a broad area of scientific study that encompasses techniques like machine learning as well as research areas like computer vision or natural language processing. AI is the effort “to create machines or things that can do more than what is programmed into them” ( Gebru, 2023). We supplement the term ‘AI’ regularly with that of technologies, thus leading to the acronym AITs. In this, we are inspired by the critical public policy scholarship of Emma Carmel and Regine Paul who use AITs to highlight the complex, contingent, and variable insertion of ‘AI’ into the societal domain ( Carmel & Paul, 2022; Paul, 2022). As a result of its frequent, ambiguous usage, ‘AI’ is a highly divisive term that is prone to numerous uncertainties ( Afina, 2022; Holland Michel, 2023; Tucker, 2022). Problematically, the term ‘AI’ may, for example, lead us to consider machines with intelligence as equal to humans, thereby potentially triggering irrelevant sets of questions ( Gunkel, 2023). As Whittaker critically contends, the publicly reported ‘advances’ in AI research are not so much the result of technological breakthroughs but “the product of significantly concentrated data and compute resources that reside in the hands of a few large tech corporations” ( 2021, 51). Further, we speak of both AI and autonomous technologies as these are closely related and arguably function along a trajectory of increasing technological complexity. Autonomy can be defined as the “programming machines to perform some tasks or functions that would ordinarily be performed by humans” ( Scharre & Horowitz, 2015, 5). As such, a weapon system may, for example, integrate autonomous or AI technologies to perform different kinds of functions ranging from intelligence analysis to mobility and targeting, with targeting functions having, by far, garnered most attention ( Boulanin & Verbruggen, 2017, 19–35). Finally, we follow Virginia Dignum’s definition of an algorithm as “a set of instructions, such as computer code, that carries out some commands” ( 2019, 3). Algorithms are used to ‘find’ patterns in datasets and “specify how to transform a given set of inputs into a desired output” ( Mackenzie, 2015, 43).

The remainder of this essay reviews our two analytical arguments outlined above and reflects on how they can advance a new research agenda of *practical normativity/normality* for the specific purpose of studying the impact of AI technologies on normative substance in international relations. We structure the two subsequent sections to include both a review of how far literature on practical normativity/normality has come, followed by an expanded discussion of our arguments on how operational design and use at sites beyond the public eye shape normative substance, as well as normativity/normality dynamics. In a third section, we briefly reflect on what AI technologies along with forms of ‘machine agency’ signify for established distinctions between agency and structure in norm research. In a fourth and final substantive part, we sketch three future research streams that draw on the insights presented but also take these a step further towards more experimental arguments that could allow us to critically study the emerging normativity/normality of AI technologies.

### Practical normativity/normality I: practices of developing and using AI technologies as sources of norms

Sociology and political theory have long captured practices, broadly understood as patterned interactions in social context ( Leander, 2008, 18), as the micro-level building blocks of the social world. As “nexuses of doings and sayings” ( Schatzki, 2012, 14), practices make norms. On the ontological level, practices and norms are therefore inseparable, since action-relevant normative substance only manifests in practices ( Bode & Huelss, 2018; Gadinger, 2022; Neumann, 2002, 200; Wiener, 2009; Wiener, 2018). This insight also applies to cases where a legal-institutionalized norm already exists that supposedly guides practices through its constitutive quality. The practical implementation of a norm always contains an element of improvisation and with it the potential to change its meaning ( Bode & Karlsrud, 2019; Hofferberth & Weber, 2015; Huelss, 2017; Pratt, 2022; Wiener, 2018). In principle, dynamic practices can thus create new norms, maintain, and stabilize existing norms, and change the substance of existing norms. Indeed, this process may increase a norm’s legitimacy ( Wiener, 2018).

How practices maintain, stabilise, or change the substance of existing norms has already been well-covered in IR norm research as part of different sub-agendas on the resilience, robustness, and erosion of norms, focusing on deliberation, communication, and public-discursive exchange ( Barbé & Badell, 2023; Deitelhoff & Zimmermann, 2020; Lantis & Wunderlich, 2018). From the start, IR norm research has studied how norms emerge in deliberative, international forums and therefore, conceptualized normative content as produced in public debate and international negotiation. What matters in this process are language-based strategies of presenting an argument such as framing, salience, and grafting ( Acharya, 2004; Keck & Sikkink, 1998; Rosert, 2019). Indeed, “states are very verbal entities” ( Hansen, 2006, 21). As Bower argues, “multilateral negotiations serve as focal points in the crystallization of emergent norms” ( 2015, 355).

Further, both localisation and contestation scholarship has invested much attention into offering deeply theorised thinking ways of thinking about how practices generate and shape normative substance. Contestation research, in particular, departs significantly from models considering norm emergence as sequentially distinct from the diffusion of norms, acknowledging instead the “norm-generative power that materialises through contestation” ( Wiener, 2018, 11). That power is vested in the public space and is often, although not necessarily, connected to public-deliberative processes. It is thus crucial whether and to what extent actors have *access* to the forums where the constitutive debate about norms happens, thereby being able to ensure that their voices are *heard* (see Wiener, 2018). In broader IR norm research, this public-communicative access has also manifested in agency-oriented concepts such as norm entrepreneurs who “attempt to convince a critical mass of states […] to embrace a new norm” ( Finnemore & Sikkink, 1998, 895). The reverse concept of “norm antipreneurs” ( Bloomfield, 2016) likewise describes agents working through public-deliberative means.

However, Wiener’s theoretical understanding of contestation explicitly goes beyond deliberation: “while mostly expressed through language not all modes of contestation involve discourse *expressis verbis*” ( 2014, 1). She includes explicit forms of contestation, such as contention, but also implicit forms, such as the absence of practices (neglect) and practices that contradict a certain reading of a norm (negation, disregard) ( Wiener, 2014, 7). In later conceptual and empirical work, contestation scholarship has begun to explore this behavioural dimension of contestation as “contestation by means of action that affects implementation” ( Stimmer & Wisken, 2019). Yet, interestingly, even the types of practices that animate behavioural contestation are performed in and attached to some form of public space (e.g. True & Wiener, 2019, 560, 563, 570). Therefore, they become directly observable.

Despite recognising the norm-generative power of practices, norm contestation scholarship arguably does not yet engage comprehensively with the theorisations of practice offered by practice theories. From a norm contestation point of view, there might be pragmatic methodological reasons for this. Studying how norms emerge in operational practices means attempting to access processes that are not directly observable nor immediately accessible to researchers because their performance does not leave discursive trails. At the same time, Wiener’s methodological toolkit has also included exercises such as the multilogue that work via reconstructing dialogues between different norm actors, thereby creatively handling an absence of readily available material ( 2018, 63–64). However, methodologies long used by practice scholars, such as praxiographies, feature many useful insights for rendering practices that are performed outside of the public space visible via for example, participant observation or interviews, that can be usefully applied to the study of emerging normative substance in operational practices interviews ( Adler-Nissen, 2016; Bueger, 2014; Nicolini, 2013; Pouliot, 2016). Still, in the context of studying technological practices of design and development, such methodological problems are likely amplified by norm and governance scholars limited technical familiarity with AI. Yet, over the past decade, we have seen an increasing number of publications and courses that are dedicated to bridging such knowledge gaps by providing more common-sensical understandings of how AI technologies work (e.g. Crawford, 2021; Goldstaub, 2021; Russell, 2019; Walsh, 2018; Walsh, 2022).

Our arguments add to this existing body of knowledge in two ways: we broaden analytical attention (1) by *expanding the types of practices* whose performance generates/shapes/sustains normative substance beyond practices that are, in some shape or form, publicly performed, voiced, framed, or deliberated; and (2) by *expanding the social sites* where such practices are performed to sites outside of or hidden from the public eye. This *practical normativity/normality* is particularly well-suited to understanding the impact on normative substance that AI technologies in and beyond the military domain can unfold ( Bode, 2023b).

A closer look at technologies in the military domain offers useful analytical inspiration for this line of thinking. Military technologies and weapon systems are often developed and deployed over many years before they even become the subject of public debate, leading to the public-deliberative emergence of new norms that can/should regulate existing practices ( Bode, 2023b). Anti-personnel mines, for example, have been used since World War II on a wide scale, but public discussion of the practices associated with their use only began in the 1970s under the CCW, culminating in the 1997 Ottawa Treaty that banned anti-personnel landmines. A time lag between the development and deployment of military technologies and a public debate about their use is typical, as the further examples of submarines, chemical weapons, nuclear weapons, or cluster munitions show.

But even without practices performed in a public or deliberative space, other types of practices create, shape, sustain, and change normative substance. In the cases described above, these are practices associated with designing, training personnel for, and using military technologies. Practices of design, for example, anchor normative substance in AI technologies. Here, we follow constructivist STS insights, in understanding technology as fundamentally social and thus as a reservoir of social practices ( Bijker & Law, 2010). These assumptions contrast with a positivist conceptualization of technology development as part of objective-neutral processes of experimentation and problem-solving. By contrast, STS thinking allows us to conceptualise the insertion of the material in the social. This perspective re-thinks existing dualisms, such as discourse vs. materiality, in favour of a “process ontology” that understands practices as relationally co-constituted by the social and the material ( Schouten & Mayer, 2016, 311). It also reverses a conventional separation between social actors, on the one hand, and technologies as materiality, on the other hand, in favour of an interactive perspective on how these interact in performing technological practices (see also section 2). STS thinking therefore allow us to question the accuracy of the distinction between technological factuality and value-based, normative decisions.

In the design, development, and use processes of AI technologies and algorithmic systems, practices can constitute/shape normative substance in at least three ways: (1) All AI technologies are based on the generation of computer models that ‘translate’ complex social activities into a problem to be solved and set a planned goal for this purpose ( Gillespie, 2014). This process is vaguely understood to be objectivist, but instead actively encodes values and therefore types of normative substance into AI technologies. Louise Amoore has therefore critically theorised algorithms as “ethicopolitical arrangement[s] of values, assumptions, and propositions about the world” ( 2020, 6). The ways in which algorithms find patterns in the data processed is inextricably linked to affording certain features and assumptions greater weight and therefore recognition than others ( Amoore, 2020, 8). As Abeba Birhane and colleagues have found in their survey of 100 highly cited machine learning papers, values such as ‘performance’ and ‘efficiency’ significantly outrank considerations of societal need or negative potential ( 2021). In AWS, civil society organisations have investigated how the generation of computer models entails the construction of target profiles, “the set of conditions under which such a system will apply force” ( Moyes, 2019, 1). Reviewing such target profiles demonstrates how their design encodes normative choices in the AI or autonomous technologies that animate them. Current target profiles are basically particular patterns of sensor data – in the form of acoustic signatures, radar signatures, heat-shapes, or visual information – that are interpreted as a target. As such, target profiles will contain both intended and unintended targets, because a broad target profile reduces the number of false negative results that the military actors using the AWS want to avoid ( Moyes, 2019, 4–5). In this way, target profiles shape normative substance at the stage of design practices in choosing instrumental parameters such as acceptable error rates for determining targets ( Crawford, 2021, 96–97).

As the technology used in AWS grows in complexity, targeting profiles could become adaptive rather than based on fixed, encoded goals set by human designers ( Moyes, 2019, 4). Using AI technologies, the system may suggest novel targets gleaned, for example, based on correlating multiple current forms of sensor data it has access to as part of a networked system with previous sensor data. This process describes a transition between different algorithms that sustain target profiles from rules-based types to generative learning types: the former are based on a designer-inserted hypothesis that the algorithm statistically tests, while the latter type experimentally constructs rules and concludes which of them were noteworthy ( Amoore, 2020, 12). When it comes to military decision support, systems that enable precisely these practices have already been used on the battlefield. As public sources reported, Ukrainian military commanders use the “AI Platform for Defense” (AIP), an AI-assisted software produced by Palantir, to provide them with “likely scenarios for the course of the war and […] spit out automated field moves” ( Fischer, 2023; see also Hudson & Khudov, 2023; Ignatius, 2022). Thus, such design practices encode value-based decisions that are not disclosed into the targeting process and its programming.

(2) The composition of the data sets used to program the algorithms that sustain AI technologies may contain data that does not cover the problem to be solved in a representative way, i.e. it may contain bias ( Sharkey, 2018). It is a well-known adage that an AI system can only be as good as the data it is trained on. Critical studies or audits of prominent image datasets, such as Image Net, have therefore demonstrated that AI technologies replicate and augment racial and gender stereotypes and prejudices ( Bolukbasi *et al.*, 2016; Chandler, 2021; Crawford, 2021, 96–97; Hooker, 2021). Well-known examples include systematically excluding women in results for occupations or associating women with particular types of occupations only, and systems performing best in classifying light-skinned males as human ( Benjamin, 2019; Buolamwini & Gebru, 2018; Birhane, 2021a). Such datasets therefore include assumed patterns of normality that become the basis for and amplified by the AI technologies that use them as training material. Many AI researchers argue that it is possible to circumvent such bias in the training data, inspiring an increasing research stream on bias mitigation strategies and their potential trade-offs ( Ferrara, 2023). To this, Amoore posits an interesting, more fundamental, two-fold critique. She holds that first, bias is an extricable part of algorithms as such systems need to contain forms of weightings to work and second, that learning algorithms are characterised by recursive, nested relations to input data rather than going through a linear sequence of steps thereby making it near impossible for humans to intervene in this non-linear process to remove bias ( Amoore, 2019, 11).

(3) Practices of using systems integrating AI technologies, including weapon systems, are typically based on certain, not openly stated assumptions, including about human-machine interactions. The literature on autonomous systems refers to such assumptions as myths ( Bradshaw *et al.*, 2013; Johnson *et al.*, 2014), while policy literature on AI talks about “technological myths” ( Natale & Ballatore, 2020) or simply common policy assumptions that need recalibrating ( Holland Michel, 2023). A persistent myth of autonomous systems is the “erroneous idea that once achieved, full autonomy obviates the need for human-machine collaboration” ( Johnson *et al.*, 2014, 58). In the context of warfare, we find this thinking present in how autonomous and AI technologies may replace human soldiers. However, the use of weapon systems integrating autonomous technologies, such as the Patriot missile defence systems, shows the opposite: introducing autonomous and AI technologies increases the complexity of tasks to be performed by human operators and demands more, not less, “human expertise and adaptative capacity” ( Johnson *et al.*, 2014, 84). Yet, such assumptions continue to shape use practices of AWS, such as the level of trust placed in system outputs or the involvement of the human operator in the decision-making loop of weapon systems ( Bode & Watts, 2021; Bode & Watts, 2023), with clear effects on normative substance.

In summary, a focus on the operational practices of designing, training for, and using AI technologies enables an analytical view on modalities and sites where norms can emerge outside of the public space and a form of public debate. This perspective can diversify how and where we can localize norms on AI. Conceiving of practical normativity/normality beyond the public space thus offers scholars of norms and governance a potentially far-reaching review of how normative space is constituted. Although we draw these insights inductively from analysing autonomous and AI technologies, they point to a broader empirical phenomenon: the initial emergence of norms in operational practices, which become objects of public deliberation or observable in the public space only at a later point in time or do not become a part of public debate at all ( Bode, 2023b).

Of course, we should not fail to recognise that such practices are still performed within a potentially dense normative structure. State actors who develop and use weapon system integrating AI and autonomous technologies, for example, do not operate in a normative vacuum but within the principles and prohibitions of international law, for example international humanitarian law (IHL) and international human rights law (IHRL). But international law provides practices with a thoroughly ambiguous, sometimes contradictory, and potentially enabling normative basis ( Hurd, 2017; Koskenniemi, 2011; Wiener, 2009). Based on existing international law, there can be very different judgments as to which practices are ‘appropriate’, and states therefore can have considerable practical and interpretative leeway ( Bode, 2023a).

### Practical normativity/normality II: the dynamic interplay between what is normative and what is normal

Through an emphasis on how practices of design, of training personnel for, and of using AI technologies performed at sites out of the public eye can become sources of normative substance, the interplay of normativity *and* normality also comes into focus. Practices can constitute ideas about which ideas of oughtness, justice, and associated moral duties apply (normativity) - but they can also make something appear normal or average behaviour (normality). So far, norm research in IR has only theorised the normality dimension of norms in a limited way and has, as a result, not examined how this dimension interacts with normativity. To do this, we can draw inspiration from IR scholarship beyond norm research. Critical security studies, such as scholarship associated with the so-called Paris School, for example, consider the emergence of *normality* within practices of exclusion and regulation ( C.A.S.E Collective, 2006). Here, understandings of the ‘normal’ arise in a process that compares, weighs, calculates and executes resulting in and based on identifying the ‘average’.

This work builds on Michel Foucault who offered an insightful theorisation of how social norms emerge beyond the law in his lectures on the *History of Governmentality* between 1977 and 1978. Foucault draws attention to the fact that the average is often considered the norm, but that this ‘norm’ is not identical with legal normativity ( Huelss, 2017). To distinguish between the two processes and deviating from everyday language terms, he referred to the effect of legal norms as a process of *normation*, whereas a process of *normalization* refers to how norms emerge in the derivation from the average such as the statical normal distribution, i.e. the Bell curve.

Foucault’s thinking offers interesting links to practice theories as the process of entrenching and spreading certain practices over time can lead to them becoming *routine* and therefore normalise practices outside of formal codification or acts of norm creation. Making something normal or normalising practices, is related to notions of “the common, the ordinary, the standard, the conventional, the regular” ( Cryle & Stephens, 2017, 1).

Normativity describes understandings of oughtness and justice, of patterns of behaviour that are morally right and just. Often, scholars argue that this normativity must be discursively expressed and established – and even publicly voiced. But this public understanding of normativity is too limiting. If we take the notion of ‘appropriateness’ that is a common feature of how norms are defined and take this to represent normativity, then what counts as “appropriate” behaviour is therefore not only defined in public deliberation, but also generated by practices in various forms. This is particularly relevant when understandings of the substance of deliberative norms collide with the normativity of practices. The normativity constructed in practices can be more impactful than what is discursively established.

The main point we want to make here is that normativity is typically conceptualised as an outcome of deliberative norm-setting. The resultant norms are thought to shaping the normality of practices. But this sequence could also be conceptualised in the reverse order where the normality of practices creates aspects of normativity. There are conceptual challenges to be tackled here: first, to develop an understanding of normativity in practices that might be different from the deliberative normativity that is central for conventional norm studies. Second, it should be further considered whether normativity is rather broadly or narrowly understood. What are the limits of ethical standards of ‘right and wrong’? Is normativity only relevant for certain types of practices? This is related to how broad or narrow we should understand norms to be and whether all norms also entail normativity. This question can be addressed in the context of a theoretical-philosophical discussion that, however, goes beyond the scope of this article. At this point, we just want to note that we find analytically beneficial to use both a broad understandings of norms (understandings of appropriateness) and a broad and relational concept of normativity. In other words, our position is that it is analytically too narrow to only focus on the most “fundamental norms” ( Wiener, 2009) such as human rights’ right to life, while leaving so-called procedural standards or similar aside and associating them with ‘lower’ overall rates of normativity. At the same time, broad norms should still be understood as entailing and informing generally elements of normativity.

As this conceptual discussion shows, understandings of normal behaviour can overlap with understandings of normative behaviour that is considered morally just or ‘right’. In fact, we consider normativity and normality to be deeply connected and inextricably linked. What is normative and what is normal is often not easily separable, but we should account for both of these dimensions in theorising how normative substance emerges – in relation to AI technologies and beyond. It then becomes an empirical question whether norms normalize practices by shaping what is appropriate in a given situation or whether practices rather normalize norms by shaping their substance based on what is established as normal. In what follows below, we concentrate on highlighting the normality dimension as this has received comparatively less analytical attention in norm research and we want to draw attention to some interesting analytical tendencies that this focus reveals. But we are conscious of the fact that this is an ideal-typical conceptualisation rather than a clear-cut empirical one.

In the context of AWS, practices associated with the design and use of AI technologies, can work to establish patterns of normality in at least two ways: (1) the algorithmic definition of parameters and target profiles at the point of design determines what counts as ‘normal’ and what counts as ‘abnormal’ or anomaly. As Amoore argues, “contemporary algorithms are not so much transgressing settled societal norms as establishing […] new thresholds of normality and abnormality” ( 2020a, 6). This encoded definition represents a clear limitation of what is conceivable as normal and abnormal in the future. AI technologies based on the technique of machine learning are particularly important to consider here, for example object recognition as part of the image-processing of computer vision. In recent years, the US military launched projects with significant financial budgets to conduct relevant research in this direction through collaborating with tech companies. Project “Maven”, initially carried out in cooperation with Google, should be mentioned here as a prime example (see Suchman, 2020a).

When examining patterns of normalisation, the use of ‘unsupervised learning’ algorithms in the context of machine learning is noteworthy. Here, algorithms aim to identify patterns of interest in the data that represent an anomaly rather than searching for patterns that it has been trained on ( Aradau & Blanke, 2018). As outlined above, “the algorithms […] generate all rules and the debate which of them was interesting” ( Amoore, 2020, 12). A widely discussed empirical example of unsupervised learning in the military context was the US National Security Agency's (NSA) Skynet programme, which endeavoured to associate movement patterns of mobile phone users based on metadata with the users’ potential terrorist activity. Controversially, this data could also be used in the wider targeting process to support target identification for drone strikes. However, public reporting showed that the system mis-identified prominent Al-Jazeera journalist Ahmad Muaffaq Zaidan in Pakistan as a member of terrorist organizations such as Al Qaeda and therefore as a potential target ( Currier *et al.*, 2015; Robbins, 2016). The travel and movement patterns associated with his investigative work apparently generated an *anomalous* or suspicious movement profile ( Aradau & Blanke, 2018; Bode & Huelss, 2021; Huelss, 2020). The processing and use of various form of digital data is part of common practices performed by the US military, as confirmed by the National Security Agency (NSA). The former director of the NSA and CIA, retired US Air Force General Michael Hayden, explicitly underlined: “We kill people based on metadata” (cited in Scahill & Greenwald, 2014). This is an example where digital normality detection may prevail over deliberative normativity considerations. In other words, what machine-learning algorithms identify as an anomalous and therefore interesting data pattern can have very immediate consequences for life and death decisions with high normative salience. Identifying a data anomaly as something abnormal that stands outside of normativity and needs to be normalized, brought in line with the normal, or rather eliminated, is central to this process that animates how technological, operational practices may shape norms. While the kill decision is not automated in scenarios such as the Skynet programme tests, the insertion of algorithms into targeting decision-making changes the quality of control that humans exercise therein. This development is therefore a decisive part of potentially normalising machine induced norm emergence that can limit human control over the use of force ( Bode & Watts, 2021; Bode & Watts, 2023; Bode, 2023b).

(2) Practices associated with the design and use of AI technologies can also ‘normalize’ by informing and influencing human actors as part of human-machine interaction. Regardless of the extent to which the “machine” makes autonomous decisions, the repeated, collective execution of a similar range of practices based on human-machine interaction over a period of time can come to be considered “normal”, increasingly “acceptable”, perhaps even desirable. In these situations, we may find an interactive exchange between normality and normativity, in which incrementally changing, collectively held understandings of appropriateness develop.

As these illustrations show, the relationship between normality and normativity remains a research-analytical challenge and requires further, detailed studies to show the possible trajectories of transition from the normal to the normative or the interaction dynamics between these two dimensions. These trajectories do not only concern how the normality of practices can influence legally codified norms as a postulated expression of normativity and make inroads into deliberation. They also raise more fundamental questions about the extent to which long-established, legally codified norms are informed and shaped by normality. “Fundamental norms” ( Wiener, 2007), such as human rights, for example, often align with dominant practices in certain cultures. Such practices typically precede a formal, legal codification. Given such possible dynamics, we could reconsider whether the effect of codified norms and the importance of deliberation are overemphasized in conventional, constructivist norm research – if such norms may correspond to normal, average behaviour anyway.

### AI Technologies, Normality/Normativity, and a Post-Human Agency

Finally, returning to the empirical field of AI technologies, we also hold that the emergence of a new, post-human actor quality in practices is one of the great challenges that contemporary norm research encounters. Distributed forms of agency between humans and ‘decision-making’ technology that arise here contrast with the established analytical categories of human actor and normative structure. Studies at the intersection of STS studies and the “new materialism” have begun to take up this question of agency ( Coole & Frost, 2010; Coole, 2013). A detailed discussion of this themes is beyond the scope of this essay, but we want to note how ‘distributed agency’ emerges in the interaction of people and the material. This has conceptual similarities to Latour’s “actant” ( 2005), but differs in defining agency capacities more narrowly ( Coole, 2013, 456–61). This form of agency does not replace human actors but is vested in the emergence of new systems where people and technologies have agency capacities in a material and a relational sense.

We should note that any conceptual notion of ‘machine agency’ cannot be clear-cut/discrete since much of it is the result of human action that has become invisible in the design process. This includes programmers who create the basic computational models and decide upon the algorithm's parameters, 'ghost workers' who prepare the data required for training machine learning algorithms through a series of micro-tasks often referred to as “labelling data”, and people whose data is used to train machine learning algorithms ( Crawford, 2021; Jaton & Bowker, 2020; Penn, 2021). A distributed concept of agency should consequently affect our understanding about what AI technologies are in the first place, as well as the type of data work they require. For prominent STS scholar Lucy Suchman, AI is therefore “a cover term for a range of technologies of data processing and techniques of data analysis based on the iterative adjustment of relevant parameters, according to some combination of internally and externally generated feedback” ( 2020b).

Moreover, the idea of distributed agency can also lead us to consider a concept of distributed normativity/normality in the context of AI and human interaction. Given that the productive substance of AI, whether it is about generative task in terms of text production or similar applications, is essentially based on a recompositing or parroting of human creative output (fed-in as learning data), it would be misleading to speak of post-human normality/normativity in the strict sense. ^9^ Nevertheless, the normative outcome of AI decision-making would still be outside of strictly human controlled normativity/normality.

Notwithstanding these qualifications, the conventional view of agency and structure in norm research might no longer do justice to such technological developments. The influential premises of structuration theory, for example, assume a logical and temporal separation of both dimensions. But what if practices performed by using AI technologies or as part of human-machine interaction override public, human agency-based deliberations of normative substance? As discussed above, machine learning may lead to the production of normative substance based on data patterns, even if such algorithmic ‘decision-making’ is no longer comprehensible and verifiable by human actors. The introduction of algorithmic ‘decision-making’ is also an onslaught on the human ability to doubt ( Amoore, 2019). The neat, seemingly objective outputs of AI that are based on a vast number of complex calculations are ultimately impossible to question in the logic of technology. Especially in the military context, but also beyond it, factors such as speed, lack of time, and high data load mean that it may no longer be possible for human operators who are in-the-loop of weapon systems to meaningfully control/understand the prompts offered by a system integrating autonomous or AI technologies ( Bode & Watts, 2021; Bode & Watts, 2023).

What is more, how machines process information, identity anomalies, produce assessment about what constitutes an ‘appropriate’ action, and execute these actions collapses an ideal-type image of control and deliberation. In other words, systems integrating AI or autonomous technologies – regardless of whether a human is monitoring the system (in-the-loop or on-the-loop) – can create normative substance through analysing sensor input at negligible time intervals, detecting deviations from “the normal”, and reacting to this deviation within the given parameters, for example by destroying the target. Therefore, machines are becoming *de facto* agents who create, maintain, or change forms of normative substance through performing practices. The resulting blend between practical normality/ normativity is a new dimension that should receive more attention in IR norm research. It may also result in fundamentally reconsidering how stable norms are over time: practical normality/normativity emerges in highly dynamic processes that no longer correspond to the idea of a changeable but largely stable normative structure.

## Future theorising on AI technologies and norms

The conceptual concerns raised, and arguments presented above lead us towards sketching several additional research streams for theorising how AI technologies may shape norms. These avenues are preliminary but are intended to give a sense of further/future direction for issues that need to be further addressed if we are grasp the potential ramifications of practical normality/normativity related to AI technologies in an IR context.

### Relationship between operational and deliberative processes of norm emergence

One of the fundamental tenets of our argument is to point IR norm research in the direction of more closely considering, conceptualising, and analysing operational practices performed outside of the public space as sources of normativity/normality. With this theoretical move, however, we do not want to dismiss or side-line public-deliberative processes of norm emergence that happen in manifold forms in the context of AI technologies. In fact, such public-deliberative processes continue to diversify across state, regional, and global levels and may thereby result in an increasingly “thick” network of written principles, guidelines, standards, or regulations interacting with each other ( Maas & Villalobos, 2023; Tallberg *et al.*, 2023). As table 1 illustrates, in 2023 alone, stakeholders started at three new, longer-term cross-regional and international public-deliberative processes and associated governance initiatives on AI technologies in the military domain. While such public-deliberative processes are moving slower in comparison to the pace and track record associated with actors designing, developing, and employing AI technologies, we can therefore expect them to produce a larger number of and more substantive outputs in the coming years.

**Table 1.** *Governance initiatives on AI technologies in the military domain starting in 2023*.

Responsible AI in the Military Domain (REAIM) Summits, co-led by the Netherlands and the Republic of Korea, first multi- stakeholder summit held in February 2023
Political Declaration on the Responsible Use of AI in the Military Domain, led by the United States with currently 51 states participating
General Assembly First Committee (Disarmament and International Security) Resolution on Lethal Autonomous Weapons Systems (LAWS) adopted 164/8/5 in November 2023 (UN Document No. A/C.1/78/L.56), starts a process to report challenges and concerns raised by LAWS from humanitarian, legal security, technological, and ethical perspectives

It is important, then, to think about the public-deliberative and the operational processes not in isolation of each other but consider how the precise dynamics of their interaction can shape that emergent norms therein take via their respective constitutive practices. We started to sketch the interaction of these processes in our previous research on emergent normativity in the context of weapon systems integrating autonomous and AI technologies. Here, we distinguish between four potential interaction dynamics that are drawn inductively from arms control cases: negative acknowledgement, positive acknowledgement, collision, and (wilful) ignorance ( Bode, 2023b, 995–96).

(1) Negative acknowledgement describes a process where actors involved in a public-deliberative process draw attention to the adverse impacts of normativity/normality originating in operational practices. Here, we would expect potential change in the normality/normativity that is emerging from practices as practices become the subject of public scrutiny, practitioners may rethink their performance of practices either as a result of awareness-raising or of new regulation (in whatever form) that specifically addresses the adverse impact of current practices. The scenario of negative acknowledgement therefore describes an interaction pattern where what is produced in the public-deliberative process has the highest potential shaping effect on the kinds of norms that emerge in the context of AI technologies. At the same time, the extent to which even specific public-deliberative outcomes in the form of guidelines etc change practices performed in operational context depends on a number of key factors, chief among them whether such agreements are legally binding nature, as well as their verification and implementation strategies.

(2) As part of positive acknowledgement, by contrast, actors involved in a public-deliberative process, consider and confirm existing practices and the normality/normativity that they constitute as “appropriate” and in line with (current) demands of the international normative framework. In this case, we would expect their public discussion not to change what kind of operational practices are performed and the associated normativity/normality. By contrast, their positive acknowledgement could instead be expected to lead to practitioners doubling down on such practices as they have now been publicly sanctioned as normal/normative.

(3) Collision describes a complex dynamic where contradictory practices constituting diverging normalities/normativities co-exist. Public deliberations point to the adverse consequences of (currently performed) practices in terms of constituting normativity/normality, attempt to address this situation through setting up a new normative framework and thereby to reconstitute normality/normativity. But this novel framework does not fully re-constitute normality/normativity as states continue to perform divergent practices that can be associated with the previously established normativity/normality. As such, a scenario of collision is a likely result when you have different international normative frameworks, including in the form of legal regulations, that different, potentially intersecting but also exclusive groups of states have signed and ratified. An example is the international normative framework on nuclear weapon systems that features two main sets of legal documents constituted by and producing colliding sets of practices: the 1970 Treaty on the Non-Proliferation of Nuclear Weapons and the 2021 Treaty on the Prohibition of Nuclear Weapons.

(4) A final interaction dynamic consists of actors in the public-deliberative process simply not speaking to or raising the emergent normativity/normality that is constituted by operational practices. Here, while the public-deliberative and the operational processes of norm emergence continue to run in parallel to each other, but do not appear to influence one another, at least not in direct ways. In such a set-up, the kind of operational practices that practitioners perform are not likely to change – at least not in the dramatic way that may result from negative acknowledgement.

These four interaction dynamics are not mutually exclusive. This means that we could see several of these dynamics at play in relation to operational and public-deliberative processes in the same policy field, e.g. the governance of AI technologies. Likewise, interaction dynamics in one policy field can change over time.

### Landscape of actors whose practices map shape normativity/normality on AI technologies

Norm research has prominently focused on a comparatively limited group of actors as major agents shaping normality/normativity. This group chiefly includes civil society actors at international and national levels, international organisations, and state representatives, often acting in diplomatic settings. Our line of thinking about how operational practices of designing AI technologies can shape normativity/normality puts the focus on another set of actors: tech companies. Scholars of private authority have long acknowledged the influence of such private, corporate actors on global governance ( Cutler *et al.*, 1999; Hall & Biersteker, 2002; Leander, 2010). Like the public-deliberative focus of norm research, corporate actors are described as having regulatory agenda-setting power through their participation in various, more or less formalised, international fora. But the role that tech companies play in shaping normativity/normality around AI technologies is, at the same time, more direct and less visible. Representatives from these tech companies are invited as recognised experts to discuss potential, public-deliberative governance solutions for AI technologies ( Bode & Huelss, 2023). But these tech companies also perform the operational practices of development and design that shape normality/normativity inherent to AI technologies in the first place.

This insight calls for reconsidering the landscape of relevant actors to study when norm scholarship wants to trace emerging normativity/normality around AI technologies. There is an emerging stream of studies who have started this endeavour, including a larger-scale research project theorising the significance of the platform nature of these companies. So far, the focus has mostly been on tracking the significant influence of “Big Tech”, such as Google ( Hoijtink & Planqué-van Hardeveld, 2022; Planqué-van Hardeveld, 2023). This denominator describes the five major tech companies that dominate the overall AI landscape in terms of the infrastructure needed to train learning algorithms in terms of data sets and data centres, the personnel with the requisite professional backgrounds needed to conduct such operations, and the output produced in the form of AI technologies. But the rise of tech companies such as OpenAI demonstrates that scholars need to cast their analytical net wider to account for the precise role of tech companies. Further, the example of AI technologies in the military domain demonstrates a changing ecosystem of companies: while governmental defence actors have initially primarily sought cooperation with the Big Tech and such defence contracts continue, over the past 2–3 years, we have seen a growing number of smaller, specialised, and often new tech companies who focus on producing AI technologies for the military domain. Such technologies are especially usable in the area of decision support. There is a lot to monitor here in terms of the roles of such tech companies versus the roles of political actors in shaping what governs AI technologies and what kind of normativity/normality these technologies produce, and how the dynamics of the evolving AI ecosystem with its growing diversity of tech companies producing AI technologies comes into play/this.

### Direction of political engagement for critical International Relations scholarship

Finally, we close with reflecting on where these analytical observations and research streams about normativity/normality in the context of AI technologies lead us as critical theorists of International Relations. Our understanding of critical IR scholarship extends beyond the Critical Theory associated with the Frankfurt School. We share Robert Cox’ basic distinction between problem-solving and critical theories famously summarised in the insight that “theory is always for someone, and for some purpose” ( Cox, 1981, 128). For us, this manifests in the desire to sustain real-world transformative impacts through our theorising based on a broad recognition of dynamics of redistribution and recognition ( Fraser, 1985). Based on this attitude, we want to add two comments about our key argument that operational practices related to designing, but also training people for using, and employing AI technologies shape normativity/normality – potentially at the expense of how normality/normativity is shaped as part of public-deliberative processes.

First, this argument points to how processes that typically run under the analytical radar should receive much closer attention and scrutiny. This should also make such operational practices a site of political engagement for both social scientists monitoring such processes closely and for the natural scientists involved in design and development. Among parts of the scientific community commonly involved in design processes of AI technologies there is a growing recognition of their responsibility manifesting in the notion of value-sensitive design ( Friedman *et al.*, 2013). Specifically, this covers not only to the design phase but also extends to the ethical consequences of using technologies in particular contexts as well as their potential long-term impacts ( Cawthorne, 2022; Cawthorne, 2023). Beyond recognising the potential normative/normal impacts of operational design and use practices, responsible research and innovation models focus on following the four principles of anticipation, inclusion, reflexivity, and responsiveness ( Stilgoe *et al.*, 2013). This thinking around value-sensitive design that rejects neutral understandings of technology and recognises its normal/normative dimension also lends itself to interdisciplinary cooperation between the social and the natural sciences.

Second, the very insight that operational practices may contravene public-deliberative processes does not mean that we should abandon public-deliberative processes. Instead, such processes remain vital for drawing public attention to the potential adverse forms of normativity/normality that take shape in the context of operational practices. As we described about, negative acknowledgement of the normative/normal consequences of such operational practices can start of process of changing such practices and thereby the normativity/normality they shape. For that to be an option, any negotiation or drafting processes of soft or hard law on AI governance need to be either already accompanied by or followed by thorough thinking around their implementation. As we noted before, the indeterminacy of international law typically defers resolution elsewhere. This “elsewhere” is particularly important in the case of AI technologies because of what we noted about practices of design, development, training, and usage. Yet, the outcomes of some of the emerging governance initiatives on AI technologies remain at the level of abstract, ambiguous principles. Just to offer one example, the Bletchley Declaration by countries attending the AI Safety Summit in November 2023 calls for the development of AI “in such a way as to be human-centric, trustworthy and responsible” ( Government of the UK, 2023). But there is no concrete pathway that would take such broad principles to the level of operational practices and thereby actually making a difference in terms of how AI technologies are developed, designed, and used ( Nadibaidze, 2023). This requires thinking in very concrete ways about translating such principles in operational terms. Such a process could be accompanied by international standards organisations such as the IEEE SA or the ISO and will require a lot of additional attention to detail.

## Conclusions

In this essay, we reflected on practices related to AI technologies that are performed beyond public deliberation and at sites beyond the public eye as sources of normativity and normality. Empirically inspired by our empirical research agenda investigating the integration of AI and autonomous technologies in weapon systems, we argue that these dynamics pose several conceptual challenges, but also opportunities, for IR norm research. These include the relationship between the emergence of norms in operational practices and in a public space, the interplay between normativity and normality, and ultimately, how the increasing spread of AI and autonomous technologies in various social settings leads to the emergence of new, post-human agency qualities with a potential effect on normative substance. Accepting these challenges offers IR norm research a broader analytical view of how and by what means international normative space is constituted. Ultimately, these challenges also offer opportunities for constructing new analytical arguments that can contribute to a better understanding of human-machine interaction and its societal consequences, thus addressing questions that are socially and politically relevant far beyond the disciplinary boundaries of IR.

## Data Availability

There is no data associated with this article.
