# Supplementary material for: Artificial Intelligence Technologies and Practical Normativity/Normality: Investigating Practices beyond the Public Space
Source: Open Res Eur. 2024 Mar 11;3:160. Originally published 2023 Sep 22. [Version 2] doi: 10.12688/openreseurope.16536.2 (PMC11015116; doi:10.12688/openreseurope.16536.2)
Supplement: Peer review history

# Reviewer report: Reviewer response for version 2

**DOI:** 10.21956/openreseurope.18650.r39587

**Published:** 2024-05-28

**Authors:** Max Lesch

**Affiliations:** 1 Peace Research Institute Frankfurt, Frankfurt, Germany

Thank you for giving me the opportunity to review the essay for *Open Research Europe*, entitled “Artificial Intelligence Technologies and Practical Normativity/Normality: Investigating Practices beyond the Public Space” by Ingvild Bode and Hendrik Huelss. The article explores the normative dimension of governing artificial intelligence (AI) technologies. The authors contribute to this vivid field in international relations by providing a practice-based approach to international norm dynamics surrounding the rapid development of AI technologies in various areas, including autonomous weapons systems. Based on their concept of “practice normativity/normality”, they provide an analytical framework for studying how the development and use of AI technologies shape international norms. This framework is based on the distinction (and interlinkage) between normality and normativity. Whereas normality connotes “the typical and average” (in practice), normativity refers to “principles of oughtness and justice”. Rather than taking international negotiations as the starting point for analysing the development of international norms, they propose to begin with the practices and agency of tech firms that develop and deploy AI technologies. The essay provides an excellent contribution and comprehensive overview of the challenge that AI technologies pose for international relations, both politically and theoretically, and promises an exciting future research agenda on AI. In the remainder of this review, I will focus on the link between normality, deviance, and normativity, and, closely related, the concept of practice, where I believe conceptual clarification could further strengthen the central argument –particularly with regard to the norm-generative effects of practice.

 How does practice become normatively meaningful? After all that is what the authors set out to study: “how artificial intelligence may shape international norms” (p. 1), which they define in line with conventional IR norms research as “understandings of appropriateness” (p. 4). Their central argument is that international norms on AI are currently mainly driven by AI practices themselves, which have been understudied in IR norms research due to a bias to focus on public deliberations. Therefore, as I understand the authors, the normality created in practice by AI technologies is the more salient source for shaping international norms and/or normativity than the public deliberations. In my view, it would help to strengthen this argument if it was more clearly distinguished from – ultimately also practice-based – approaches in other theoretical traditions focussing on the norm-generative effects of for example norm violations, deviance and precedents. Here, the absence of normality or its rupture is the primary norm-generative factor.

 This also points to a more fundamental conceptual issue: When we take norms as “understandings of appropriateness” and, very similarly, normativity as “principles of oughtness or justice”, then I think it becomes difficult to see normativity (or norms) in normality. Normativity is only induced when these practices, this normality, are actualised as appropriate – or inappropriate. As Christoph Möllers (2020, 66) argues norms are more than representations of normality or regularity. From this perspective, regularity may even be devoid of normativity because we usually recognise and negotiate what is appropriate in the face of what is not normal, abnormal – or deviant. This point is of course recognized by Bode and Huelss, when they discuss “negative acknowledgements” or “collision” in their analytical framework (p. 11), but it could be made more explicit with regard to the underlying concept of norms and normativity as well as regarding the sites and actors that engage in negative acknowledgements beyond formal deliberative forums. Linking this argument to the sociology of deviance in IR (Adler-Nissen 2014), would suggest to recalibrate the metaphor of taking normality and normativity as “two sides of the same coin” (p. 4). Perhaps, normality and deviance are the two sides of that coin – the norm – and normativity is what distinguishes them. In my view, the abnormal or deviant is conspicuously absent from the notion of ‘practical normativity/normality’, at least at first sight, but is quite important for the making, maintaining and unmaking of international norms (Lesch 2023, 3). For example, we might only see some (informal or social) norms already at play/emerging in the governance of AI at the moment they are violated and labelled as such. In short, my suggestion would be to include the “non-normal” in the concept of norms and normativity, which would also better ground the framework developed on p. 11. 

 That being said, a focus on norm violations does of course not help empirically if we cannot observe negative reactions to norm violations or the labelling of deviance and stigmatization of norm breakers. While I wonder to what extent the discourse includes such responses – and the authors refer to some of them – the author’s argument would probably be that the more decisive practice-driven factor (in this case) is normality and its productivity for international norms. Writing as someone very sympathetic with practice-based approaches to the study of norms in IR, I would like to invite the authors to invest a little more in explaining how practices really normalise? Where can we see the performative effects of practices on norms (or norm change) and how can we analytically capture this normativity? This also speaks to the agent-structure debate that the authors touch on briefly. Perhaps it would help to clarify the authors’ interpretation of Giddens’ structuration theory, which I would read slightly differently in terms of his suggestion that agency and structure are intimately intertwined through practice – in the duality of structure (Gidden 1979, 69). This is of course echoed by Antje Wiener’s (2007) notion of “dual quality” of norms in IR. In other words, when do practices become structuring and when do they not? In my view, STS approaches could help to strengthen the argument about normalisation, the role of algorithms as “actants” etc. as creating normativity by virtue of being performed – also in view to other norm development processes that evolve below the radar of formal treaty making, for instance, through expertise. But there are also cases where normality is not norm-generative and my suggestion would be to use this distinction (to non-cases) to argue when and how these practices have performative effects and shape international norms.

 Finally, and closely related, I wondered what kind of norms the authors are referring to that are shaped by international practice. The distinction between deliberative forums would suggest that they have in mind some sort of formalised or codified international treaty norms – which do not keep pace with developments on the ground. However, the definition of norms given would also refer to social or informal norms that can emerge without, before or alongside legal norms. In international law, the shift to informality is also discussed as an alternative strategy to advance law-making processes where formal treaty-making is blocked (Mantilla 2024; Reiners 2024). While this can be productive for the development of international norms, the present case would probably point in the opposite direction, where practice-based and informal norm dynamics – from a normative point of view – hinder or undermine the regulation of AI technologies. A discussion of this aspect could also provide an opportunity (if space allows) to spend a few sentences on how we can generalize from the case of AI governance to other issue areas.

Is the argument persuasive and supported by appropriate evidence?

Yes

Is the work clearly and cogently presented?

Yes

Is the topic of the essay discussed accurately in the context of the current literature?

Yes

Does the essay contribute to the cultural, historical, social understanding of the field?

Yes

Reviewer Expertise:

international norms, international practices, international law, human rights, peace and security law

I confirm that I have read this submission and believe that I have an appropriate level of expertise to confirm that it is of an acceptable scientific standard, however I have significant reservations, as outlined above.

## References

[1] : Stigma Management in International Relations: Transgressive Identities, Norms, and Order in International Society. *International Organization* .2014;68(1) : 10.1017/S0020818313000337 143-176 10.1017/S0020818313000337

[2] : From Norm Violations to Norm Development: Deviance, International Institutions, and the Torture Prohibition. *International Studies Quarterly* .2023;67(3) : 10.1093/isq/sqad043 10.1093/isq/sqad043

[3] : From treaty to custom: Shifting paths in the recent development of international humanitarian law. *Leiden Journal of International Law* .2024; 10.1017/S0922156523000705 1-20 10.1017/S0922156523000705

[4] : The possibility of norms: social practice beyond morals and causes. *Jurisprudence* .2023;14(1) : 10.1080/20403313.2022.2139966 118-126 10.1080/20403313.2022.2139966

[5] : States as bystanders of legal change: Alternative paths for the human rights to water and sanitation in international law. *Leiden Journal of International Law* .2023; 10.1017/S0922156523000535 1-20 10.1017/S0922156523000535

[6] : The Dual Quality of Norms and Governance beyond the State: Sociological and Normative Approaches to ‘Interaction’. *Critical Review of International Social and Political Philosophy* .2007;10(1) : 10.1080/13698230601122412 47-69 10.1080/13698230601122412

[7] : Central Problems in Social Theory: Action, Structure, and Contradiction in Social Analysis. *University of California Press* .

# Reviewer report: Reviewer response for version 2

**DOI:** 10.21956/openreseurope.18650.r39585

**Published:** 2024-04-30

**Authors:** Haekal Al Asyari

**Affiliations:** 1 University of Debrecen, Debrecen, Hajdú-Bihar, Hungary; 2 Universitas Gadjah Mada, Yogyakarta, Special Region of Yogyakarta, Indonesia

The topic of the essay is accurate with the context of the current literature. The research proposed a rather obvious but often overlooked concept on how operational practices of technology companies contribute towards AI norm-making process. The take on interplay between normality and normativity as the basis of analysis adds value to the discourse of AI governance.

 While it seems that the object of the research is regarding autonomous weapons systems, the authors does not seem to incorporate the discussion of AWS throughout the introduction consistently. Particularly throughout the first half of the essay, the narrative on the concept of AI implies a general example of AI technologies in the everyday social life. Furthermore, the statement of ‘ *AI technologies that are performed by actors at **site outside of the public eye***’ does not provide a direct correlation to the subject of AWS. Perhaps it would be better to further elaborate on the meaning of ‘site outside of the public eye’.

 The authors have provided an extensive explanation on the definition of AI. An eye-catching statement in that section states ‘… *both AI and autonomous technologies as these are closely related and arguably function along a trajectory of increasing technological complexity*’. It is arguable whether all AI technologies are autonomous. Perhaps providing a reference would add credibility to the statement.

 The essay provides thorough explanation on how the practices of designing and using AI technologies that are performed by public actors in the military field, have become a phenomenon of normality, prior to the normative progress that shapes the existing legal framework on AI governance. The authors have done a great job at explaining the concepts of design and development proves of AI technologies and algorithmic systems. It is made clear from such examples, where the basis of ‘normality’ comes from.

 It is very interesting to understand the proposed ‘reverse order’ conceptualization of norm setting which comes of normality of practices. The authors have tackled this challenge by addressing two main points; developing an understanding of normativity in practices that might be different from the deliberative normativity, and by considering whether normativity is broadly or narrowly understood.  

 All in all, the research is conceptually thought provoking and opens a broad horizon for future research; as it is intended.

Is the argument persuasive and supported by appropriate evidence?

Yes

Is the work clearly and cogently presented?

Yes

Is the topic of the essay discussed accurately in the context of the current literature?

Yes

Does the essay contribute to the cultural, historical, social understanding of the field?

Yes

Reviewer Expertise:

Cyberspace Law, Cyberspace and AI Governance

I confirm that I have read this submission and believe that I have an appropriate level of expertise to confirm that it is of an acceptable scientific standard.

# Reviewer report: Reviewer response for version 2

**DOI:** 10.21956/openreseurope.18650.r38848

**Published:** 2024-04-15

**Authors:** Marijn Hoijtink

**Affiliations:** 1 Department of Political Science, University of Antwerp, Antwerp, Belgium

I have now had the opportunity to review the revised article. I am pleased with the authors’ detailed response to my comments and the revisions made.

Is the argument persuasive and supported by appropriate evidence?

Partly

Is the work clearly and cogently presented?

Partly

Is the topic of the essay discussed accurately in the context of the current literature?

Yes

Does the essay contribute to the cultural, historical, social understanding of the field?

Yes

Reviewer Expertise:

Critical security studies, Science & Technology Studies, military AI and modern warfare

I confirm that I have read this submission and believe that I have an appropriate level of expertise to confirm that it is of an acceptable scientific standard.

# Reviewer report: Reviewer response for version 2

**DOI:** 10.21956/openreseurope.18650.r39578

**Published:** 2024-04-12

**Authors:** Damián Tuset Varela

**Affiliations:** 1 University of Jaén, Jaén, Spain

The article under review presents an insightful analysis into the evolving landscape of artificial intelligence (AI) governance, particularly emphasizing the critical role operational practices play in shaping norms related to AI technologies. Through an interdisciplinary approach that melds international relations (IR), norm research, and science and technology studies (STS), the authors argue compellingly that the rapid advancements in AI have outpaced traditional public-deliberative governance processes. They propose that operational practices, especially those carried out behind closed doors by private tech companies in the design, development, and deployment of AI technologies, are pivotal in the formation of AI norms, including those concerning military applications.

 Expanding on this, the paper delineates four distinct dynamics that characterize the interaction between public-deliberative processes and operational practices: negative acknowledgement, positive acknowledgement, collision, and ignorance. This nuanced analysis challenges the existing norm research narrative that predominantly focuses on state and civil society actors, calling instead for a broader lens that includes the influential role of tech companies in shaping normativity around AI technologies.

 However, to ensure the article's scientific soundness and enhance its impact within the scholarly community and beyond, several areas require further attention and development. Firstly, the conceptual framework, particularly the distinction between normativity and normality, needs clearer definition and illustration. This will not only strengthen the paper's foundational arguments but also make the complex dynamics of AI governance more accessible to readers. The inclusion of concrete empirical examples demonstrating how operational practices in AI design and development influence norm formation would significantly bolster the paper's arguments, providing tangible evidence of the theoretical propositions put forth.

 Moreover, a more balanced analysis that engages with counterarguments regarding the efficacy of public-deliberative processes in governing AI would enrich the discourse. Exploring perspectives that underscore successful instances where these processes have effectively shaped norms and regulated AI technologies could offer a counterbalance to the paper's emphasis on operational practices. Additionally, the article would benefit from an expanded discussion on policy implications. Articulating detailed recommendations for policymakers on developing adaptive and responsive governance frameworks capable of addressing the ethical, legal, and societal challenges posed by AI is crucial.

 By addressing these points, the article can significantly enhance its contribution to the field, offering a more comprehensive and nuanced understanding of AI governance and the interplay between operational practices and norm formation. This would not only solidify the paper's scientific soundness but also underscore its relevance and applicability to a broad spectrum of stakeholders engaged in navigating the complexities of AI technologies and their governance.

Is the argument persuasive and supported by appropriate evidence?

Yes

Is the work clearly and cogently presented?

Yes

Is the topic of the essay discussed accurately in the context of the current literature?

Yes

Does the essay contribute to the cultural, historical, social understanding of the field?

Yes

Reviewer Expertise:

Artificial Intelligence ; International Relations; Diplomacy ; Cybersecurity, UE Law

I confirm that I have read this submission and believe that I have an appropriate level of expertise to confirm that it is of an acceptable scientific standard.

# Reviewer report: Reviewer response for version 1

**DOI:** 10.21956/openreseurope.17850.r35278

**Published:** 2023-11-08

**Authors:** Marion Laurence

**Affiliations:** 1 Department of Defence Studies, Royal Military College of Canada, Toronto, Ontario, Canada

This article uses international practice theory and evidence from the field of artificial intelligence technologies (AIT) to analyze shortcomings in the literature on norm emergence and norm contestation. The authors distinguish between the concepts of *normativity*, defined in terms of justice and ‘oughtness,’ and *normality*, understood in terms of ideas about what is ‘standard’ or ‘average.’ They build on this distinction to put forward a research programme on AI and practical normativity/normality, which has two main pillars. First, they argue that operational practices of designing and using AI can make norms, even though they are typically performed outside the public domain. This focus on private norm-making serves as a corrective to much of the norms literature, which focuses disproportionately on public discourse and deliberation. Second, they argue that the interplay between normality and normativity is analytically influential in the making of norms, and that this has under-examined effects on human agency. If practices can transform and constitute norms, then normality shaped by AI technologies can lead to “non-human generated normativity,” (p. 1) a possibility not currently accounted for in norms research and one with important implications for efforts to regulate AIT.

**Report**

 The article provides a succinct overview of trends in the research on norm life cycles and norm contestation, including an accurate discussion of current debates in IR theory and gaps in the scholarly literature. The authors rightly point out that existing norms research has focused primarily on public deliberative processes (often in multilateral settings) while neglecting norm emergence and contestation that may occur outside the public eye (see also Stimmer & Wisken 2019). There is one additional point from the literature that might be worth noting: some practice theorists subscribe to a normative conception of practice that defines normativity very broadly (e.g. Rouse 2001, 2007; cf. Bernstein and Laurence 2022). In my view, the authors’ distinction between normativity and normality is theoretically sound and analytically helpful, but others might disagree because they effectively collapse normativity and normality into one category.

 The article’s central argument is clear and well presented. It also provides an accessible introduction to debates about artificial intelligence technology and autonomous weapons systems for non-specialists. Indeed, the concept of non-human generated normativity is compelling, if somewhat alarming, for those not familiar with the field of AI. It raises important question about evolving dynamics of norm contestation and human agency in global governance.

 The argument is original, persuasive, and well-supported by evidence, mostly drawn from the area of AI and autonomous technologies that are used in weapons systems. The focus on norm emergence and contestation outside the public eye is timely. The authors observe, correctly, that normativity is produced in and through practice (see also Wiener 2004; Krook and True 2010). This means that the concept of “normality,” especially as used by AITs can easily have normative implications, with targeting algorithms for autonomous weapons systems being just one example (see p. 6-7). The authors also note that AIT can replicate and amplify existing forms of normativity, which are embedded within them by programmers (e.g. reinforcement of racial and gender stereotypes, see p. 6-7). This makes me wonder about the degree to which ‘new’ types of AI-generated normativity are derived from pre-existing norms. It seems like they are, at least initially, products of interactions between AIT and human inputs, but products that can take of a life of their own, so to speak. The authors seem aware of this, but it may be worth underlining the human roots of “post-human agency” and AI-generated normativity, if only to emphasize the importance of thinking carefully about what norms and assumptions humans choose to embed within AIT to begin with (p. 9).

 The article makes important contributions to the fields of IR theory, global governance, and the literature on norm contestation. It also improves IR scholars’ understanding of artificial intelligence technologies and global efforts to regulate them. In terms of broader relevance, I wonder about the extent to which norm scholars’ tendency to focus on public deliberation instead of private settings is an artefact of methodological decisions related to access (or lack thereof) to research sites and what can be easily observed or measured (noted on p. 5). In the field of AI, this is likely compounded by a lack of technical expertise among norm scholars. It might be interesting to expand on this methodological motivation in future work.

Is the argument persuasive and supported by appropriate evidence?

Yes

Is the work clearly and cogently presented?

Yes

Is the topic of the essay discussed accurately in the context of the current literature?

Yes

Does the essay contribute to the cultural, historical, social understanding of the field?

Yes

Reviewer Expertise:

International relations theory; global security governance; political sociology of international organizations.

I confirm that I have read this submission and believe that I have an appropriate level of expertise to confirm that it is of an acceptable scientific standard.

## References

[1] : Two Concepts of Practices. In: Schatzki TR, KK Cetina and E von Savigny (eds) The Practice Turn in Contemporary Theory. *Routledge* .2001;198-208

[2] : Social Practices and Normativity. *Philosophy of the Social Sciences* .2007;37(1) : 10.1177/0048393106296542 46-56 10.1177/0048393106296542

[3] : "Practices and Norms: Relationships, Disjunctures, and Change," in Conceptualizing International Practices: Directions for the Practice Turn in International Relations, ed. Bueger, C., A. Drieschova and T. Hopf. *Cambridge: Cambridge University Press* .2022;

[4] : The dynamics of dissent: when actions are louder than words. *International Affairs* .2019;95(3) : 10.1093/ia/iiz019 515-533 10.1093/ia/iiz019

[5] : Rethinking the life cycles of international norms: The United Nations and the global promotion of gender equality. *European Journal of International Relations* .2012;18(1) : 10.1177/1354066110380963 103-127 10.1177/1354066110380963

[6] : Contested Compliance: Interventions on the Normative Structure of World Politics. *European Journal of International Relations* .2004;10(2) : 10.1177/1354066104042934 189-234 10.1177/1354066104042934

# Response

**Published:** 2024-02-01

**Authors:** Ingvild BODE

We want to start by expressing our appreciation for this constructive and insightful review. We have enjoyed reviewing our article again in light of your comments and engaging with them has considerably strengthened this revised version of our article.   We have taken all comments on board which has added further depth to our argument. In particular, we now reflect much more explicitly on the dynamic interplay between normativity and normality. Further, we have separated our thinking on post-human agency by putting this into a dedicated section and reflecting more explicitly on where ”machine agency” comes from and how it still remains tied to human agency. We also added an argument about “distributed normativity” here. Finally, we have added further clarification about the methodological problems that studying processes of designing AI technologies raises.

# Reviewer report: Reviewer response for version 1

**DOI:** 10.21956/openreseurope.17850.r35276

**Published:** 2023-11-02

**Authors:** Marijn Hoijtink

**Affiliations:** 1 Department of Political Science, University of Antwerp, Antwerp, Belgium

This essay brings an original and important perspective to the study of the emerging governance of AI technology, specifically within the field of defense. Based on an extensive discussion of existing scholarship on norms in International Relations, the essay argues that even if formal deliberations of norms related to military AI appear to be highly contested and slow, this does not mean that military AI is unregulated or that there is an absence of norms in this field. Rather, as the authors argue, in this context, norms are still produced in the operational practices of designing and using AI technologies. This is an important argument analytically, politically and (potentially) normatively. Yet, I do also think that the argument can be pushed in a number of ways, specifically when it comes to (1) the analytical distinction between normativity and normality; (2) the interaction between formal and deliberative processes of norm development (the ‘front stage’) and the practical development of norms (the ‘back stage’ of norm development); (3) the politics involved in the production of norms and normality; and (4) the normative implications of the practical development of normativity/normality.

 First, related to the distinction between normativity and normality that the authors draw, I wonder if that distinction can and should be drawn in support of the authors argument. Looking at the examples that were given under sections I and II, it was not fully clear to me why these were examples that supported only normativity *or *normality. More fundamentally, it seems to me that normality necessarily comes into play when normativity is defined in the way the authors do, e.g. as emerging in and through operational practices of design and development. Perhaps this is what the authors have in mind when they refer to the “interaction” between normativity and normality, but this should have been clarified more: are they two sides of the same coin? Do they have a different temporal register? In this context, it is not so helpful that the authors fall back on a more formal, legal or deliberative understanding of normativity when defining normality (in opposition to normativity it seems). Again, I assume that this is not the point they would like to make, but the fact that they distinguish between normativity and normality in different sections and provide different examples to support each aspect of norm development raises these questions.

 Second, in addition to the interaction between normativity and normality, I would like to know more about the interaction between the formal and deliberative processes of norm development and the practical and operational context. How do the authors assess the relevance of those formal deliberations that are still happening (they even seem to proliferate), albeit in a very slow manner? Can some of the ‘back stage’ discussions and deliberations at the formal international governance stage not be part of the production of normativity/normality as the authors describe it? Where to draw the line? Or is there something very specific about the technical/operational context of design and development?

 Third, and related, I would like to push the authors to be explicit about the political stakes involved in the production of normativity/normality within an operational context of design and development. The authors mention the role of new tech companies and the ways in which through their platforms and models, specific values and value-based decisions become inscribed into the technology, but I would like to know how to square this against other (public) actors involved in the development and deployment of AI technology. *Whose* values become inscribed, especially within a military context?

 Fourth, and finally, I would like to encourage the authors to be more outspoken about their own into their own normative position or preferred ways forward. Where and how should norm development in relation to military AI take place? Are deliberative (democratic) processes leading to more formal rules and principles still the most ideal outcome, even if these are inevitably slow and potentially outpaced by the development of the technology? Or does the essay in the end call for more political engagement with designers and the design process. Where should our political engagement lie?

Is the argument persuasive and supported by appropriate evidence?

Partly

Is the work clearly and cogently presented?

Partly

Is the topic of the essay discussed accurately in the context of the current literature?

Yes

Does the essay contribute to the cultural, historical, social understanding of the field?

Yes

Reviewer Expertise:

Critical security studies, Science & Technology Studies, military AI and modern warfare

I confirm that I have read this submission and believe that I have an appropriate level of expertise to confirm that it is of an acceptable scientific standard, however I have significant reservations, as outlined above.

# Response

**Published:** 2024-02-01

**Authors:** Ingvild BODE

We want to start by expressing our appreciation for this constructive and insightful review. We have enjoyed reviewing our article again in light of your comments and engaging with them has considerably strengthened this revised version of our article.   We have taken all comments on board which has added further depth to our argument. In particular, we now reflect much more explicitly on the dynamic interplay between normativity and normality. We completely agree with your suggestion to characterise these dimensions as inseparable in practice and as two sides of the same coin. In response to your comments about the precise relationship between public-deliberative processes and operational practices in shaping normativity/normality we have added an additional section at the end of the essay entitled ”future theorising on AI and norms”. This covers both this point, as well as the important questions you raised about actors beyond states and where our political engagement should lie.
